# Multi-Level System to Assess Toxicity in Water Distribution Plants

**DOI:** 10.3390/ijerph19148469

**Published:** 2022-07-11

**Authors:** Gabriele Magara, Katia Varello, Paolo Pastorino, Danila Raffaella Francese, Paola Arsieni, Marzia Pezzolato, Loretta Masoero, Erika Messana, Barbara Caldaroni, Maria Cesarina Abete, Sabina Pederiva, Stefania Squadrone, Antonia Concetta Elia, Marino Prearo, Elena Bozzetta

**Affiliations:** 1 Department of Chemistry, Biology and Biotechnology, University of Perugia, 06123 Perugia, Italy; magara.gabriele@gmail.com (G.M.); bcaldaroni@gmail.com (B.C.); antonia.elia@unipg.it (A.C.E.); 2 Istituto Zooprofilattico Sperimentale del Piemonte, Liguria e Valle d’Aosta, 10154 Turin, Italy; katia.varello@izsto.it (K.V.); paola.arsieni@izsto.it (P.A.); marzia.pezzolato@izsto.it (M.P.); loretta.masoero@izsto.it (L.M.); erika.messana@izsto.it (E.M.); mariacesarina.abete@izsto.it (M.C.A.); sabina.pederiva@izsto.it (S.P.); stefania.squadrone@izsto.it (S.S.); marino.prearo@izsto.it (M.P.); elena.bozzetta@izsto.it (E.B.); 3 Azienda Sanitaria Locale TO3, ASL-TO3, Rivoli, 10098 Turin, Italy; francesedanila@gmail.com

**Keywords:** Calux, cyanotoxins, EPC cell line, oxidative stress biomarkers, pathogens, water concentrates

## Abstract

The toxicity of water samples from water distribution plants needs to be investigated further. Indeed, studies on the pro-oxidant effects driven by tap water are very limited. In this study, the water quality, pro-oxidant effects, and potential health risks driven by exposure to groundwater samples from two water plants (sites A and B) located in Northwestern Italy were investigated in a multi-level system. Physicochemical parameters and the absence of pathogens, cyanotoxins, and endocrine active substances indicated a good water quality for both sites. The 25 metals analyzed were found under the limit of quantification or compliant with the maximum limits set by national legislation. Water samples were concentrated by the solid-phase extraction system in order to assess the aquatic toxicity on Epithelioma papulosum cyprini (EPC) cell line. Levels of superoxide dismutase, catalase, glutathione peroxidase, glutathione S-transferase, and glutathione reductase were evaluated through the Integrated Biomarkers Response (IBRv2) index. EPC cell line was found a sensible model for assessing the antioxidant responses driven by both water concentrates. A similar antioxidant response was shown by plots and IBRv2 suggesting a muted risk for the two sampling sites.

## 1. Introduction

In the last decades, the water quality requirement has gradually increased due to the multiple needs and uses of a domestic, collective, and urban nature, as well as for agricultural and industrial demands [1]. This scenario requires a more thorough use of both telluric water resources, which are largely insufficient, and surface water [1]. Water intended for civil and agri-food use must have specific physicochemical characteristics and organoleptic properties. Indeed, a good quality of water is of crucial relevance not only for the well-being of humans and all living organisms, but also for the safety of aquatic ecosystems [2,3]. The characteristics of water often do not completely satisfy the natural requirements for potability (Council Directive 98/83/EC of 3 November 1998 on the quality of water intended for human consumption; Official Journal L 330, 05/12/1998 P. 0032-0054); indeed, metals in water intended for agri-food and human consumption must be under the limit threshold imposed by European law (Council Directive 98/83/EC of 3 November 1998 on the quality of water intended for human consumption; Official Journal L 330, 05/12/1998 P. 0032-0054) in order to avoid toxicological and ecotoxicological effects on aquatic organisms, such as oxidative stress, neurotoxicological disorders, anaemia, liver and kidney damage, pulmonary disease and stomach and intestinal irritation [4].

Moreover, due to the presence of microorganisms and organic and mineral substances deriving from natural phenomena, it is necessary to treat water to improve its quality before its use [5]. For decades, the most used water disinfection methods for civil and agri-food purposes have been performed using non-selective oxidants, such as chlorine and chlorine derivatives. The best advantage of these methods lies in the potential residual disinfection, which not only eliminates pathogenic microorganisms in a relatively short period of time, but also prevents them from reappearing along the distribution network over time [6,7]. The chemicals used for water disinfection can not only inactivate microorganisms, but also make them unable to reproduce, causing a series of irreversible damage, such as inactivation of enzymatic pathways, destruction of nucleic acids, and alterations of protoplasmic cells. However, the process may lead to the generation of by-products (DBPs), which have often shown toxic and mutagenic effects [2,8,9]. More than 600 by-products of chlorinated biocides have been identified, of which 20–30% are represented by trihalomethanes (THM) and haloacetic acids (HAA) [8,9,10,11,12,13,14,15]. The overall human and aquatic toxicity of water samples from water plants have been still poorly investigated and need to be assessed [2,16,17]. In recent decades, scientific research has focused on investigating the effects of selected chlorine compounds or DBPs on murine and fish models [18,19], rather than considering a more realistic scenario to investigate the environmental toxicity and risk. Indeed, the holistic information on the pro-oxidant effects driven by chlorinated tap water concentrates from water distribution plants is still very limited [2,16]. Oxidative stress is a physiological condition caused by the disruption of the physiological balance between the production of reactive oxygen species (ROS) and their elimination by antioxidant defense systems [20]. Any disturbances in the physiological redox state can promote toxic effects for the production of peroxides and free radicals that damage all components of the cell, including proteins, lipids and DNA. Superoxide dismutase (SOD), catalase (CAT), glutathione peroxidase (GPx), glutathione S-transferase (GST), glutathione reductase (GR), glyoxalase I and II (GI and GII) and lactate dehydrogenase (LDH) represent a key defense line against ROS in aquatic organisms when exposed to environmental contaminants [21,22,23,24]. SOD acts as a first shield against superoxide anion, CAT reduces H_2_O_2_ in the water while GPx reduces peroxidized lipids using reduced glutathione (GSH) as an electron donor. GST is a phase II enzyme that combines GSH with reactive xenobiotic to metabolize it. GR is an enzyme that ensures regeneration of the GSH reservoir from the oxidized glutathione (GSSG). Variations in the levels of these enzymes provide pivotal information on the health of organisms and their ability to respond to stressors. However, the holistic interpretation of a panel of biomarkers responses to address a potential risk driven by xenobiotics can sometimes result in being difficult and inconclusive [25]. The Integrated Biomarkers Response (IBRv2) is a powerful tool that provides an integrated and synthetic result of the biomarkers’ responses, also allowing the comparison between multiple survey sites [26]. Fish can represent reliable experimental models to investigate health risks [2,16,19,27,28,29]. Following the strict restrictions of the European Ethics Committee for scientific testing on vertebrates, fish cell lines can be considered a good substitute for in vivo models, limiting the experimental investigation on vertebrates. The Epithelioma papulosum cyprini (EPC) cell line is proved to be a good experimental model for the assessment of toxicity driven by environmental pollutants [21,30]. EPC cell line, established from proliferative skin lesions of the common carp (*Cyprinus carpio*), which is widely employed for diagnosis of fish viral diseases [31]. The aim of the study was to assess contamination and aquatic toxicological risk of tap groundwater concentrates from two sites in Northern Italy, using an integrated system with particular focus on the oxidative stress responses the EPC cell line. The prooxidant effects driven by chlorinated tap water from water distribution plants are still poorly investigated in aquatic organisms. The results obtained enrich the state of knowledge in respect of previous studies, filling the scientific gap about water security and the potential environmental risk driven by chlorinated water concentrates from water distribution plants.

## 2. Materials and Methods

### 2.1. Water Sampling and Sites

During 2021 drinking water from groundwater was sampled from two sites located in North Italy (site A and B) during summer. Both sites are small towns (4000–6000 inhabitants) with livestock and agriculture production. Water samples were collected in plastic sterile containers (25 L) at both sites nearby the livestock facilities.

### 2.2. Determination of the Main Physicochemical Water Parameters

The main physicochemical water parameters were assessed at the two sampling sites. Water conductivity (mS cm^−1^) and pH were recorded using field meters (HI 9033 conductivity meter; HI 9125 pH/ORP meter; Hanna Instruments Inc., Woonsocket, RI, USA). Water samples (*n* = 3 per site) were also collected in sterile containers, brought to the laboratory where they were immediately analyzed on a benchtop multi-parameter spectrophotometer (HI 83200-02, Hanna Instruments Inc., Woonsocket, RI, USA) according to the manufacturer’s instructions. The concentration of nitrates (NO_3_^−^, mg L^−1^) was calculated using an adaptation of the cadmium reduction method and absorption was measured at 525 nm; the ammonia content (NH_4_^+^, mg L^−1^) was calculated by adaptation of the Nessler method and absorption was measured at 420 nm [32]; phosphate concentration (PO_4_^3-^, mg L^−1^) was calculated by adjusting the ascorbic acid method [33] and measuring the absorption at 610 nm. Three replicates for each parameter were measured.

### 2.3. Metals Detection

Samples were filtered on a metal-free paper filter, 4.3 mL of water was then collected in 15 mL Falcon tubes and added with 0.7 mL of 69% nitric acid. The acidified sample was subjected to instrumental analysis after adding the internal standard (50 µL of rhodium solution, Rh, at 1 mg L^−1^). An Inductively Coupled Plasma Mass Spectrometer (ICP-MS Xseries II, Thermo Scientific, Walthamm, MA, USA), equipped with an autosampler, was utilized for the detection of aluminum (Al), antimony (Sb) arsenic (As), beryllium (Be), Bismuth (Bi), cadmium (Cd), chromium (Cr), cobalt (Co), copper (Cu), gallium (Ga), iron, (Fe), indium (I), manganese (Mn), molybdenum (Mo), nickel (Ni), lead (Pb), rubidium (Rb), selenium (Se), silver (Ag) tin (Sn), thallium (Tl), uranium (U), vanadium (V), zinc (Zn). Mercury (Hg) was quantified by the direct mercury analyzer DMA-80 (Direct Mercury Analyzer, Milestone). The DMA-80 is based on the atomic absorption spectrophotometry technique. The samples were weighed in duplicate and the vessel containing the sample was running in a furnace under a flow of oxygen, where the complete chemical and thermal decomposition of the sample takes place. The passage of mercury vapors generates an absorbance of the light signal at 253.65 nm. The limit of quantitation (LOQ) was 0.005 mg kg^−1^ for cadmium and antimony, 0.001 mg kg^−1^ for mercury, 0.010 mg kg^−1^ for the other elements. Analyses were performed in triplicates.

### 2.4. Viral and Bacterial Pathogens in Water

Tap water (50 mL) from sites A and B was tested for the occurrence of Hepatitis A virus (HAV), Hepatitis E virus (HEV) and Norovirus 1 and 2, which represent harmful pathogens for human and animal health. Five aliquots (350 µL) of water from each site were used for the viral RNA extraction. One milliliter of Trizol was added to each aliquot. Samples were incubated for 10 min at room temperature, then 280 µL chloroform was added. After incubation (15 min), samples were centrifuged at 12,000 rcf for 15 min (4 °C). The supernatants were supplemented with 700 µL isopropanol and centrifuged at the same conditions reported above. One milliliter of 70% ethanol was added to pellets and centrifuged at 7500 rcf for 5 min (4 °C).

The primers HAV68Fw (5′-TCACCGCCGTTTGCCTAG-3′), HAV240Rw (5′-GGAGAGCCCTGGAAGAAAG-3′) and the probe HAV150 (5′-FAM-CCTGAACCTGCAGGAATTA-GB-3′) for HAV detection were used as described by Costafreda et al. [34]. Primers and probe were designed to detect all HAV genotypes. The PCR assay was optimized using a total reaction volume of 25 µL. For a single reaction Ultrasense one step qRT-PCR mix (Invitrogen)1X, HAV68Fw 500 nM, HAV240Rw 900 nM, probe 250 nM, Rox reference dye 1X and 5 µL RNA extract were used. Cycling condition was as follows: 60 min at 55 °C, 5 min at 95 °C, and 45 cycles of three step cycling consisting of denaturation at 95 °C for 15 s, annealing at 60 °C for 1 min and extension at 65 °C.

Primers and probe for HEV were used as described by Jothikumar et al. [35]. The forward primer was JVHEVF (5′-GGTGGTTTCTGGGGTGAC-3′), the reverse primer was JVHEVR (5′-AGGGGTTGGTTGGATGAA- 3′) and the probe was JVHEVP (5′-FAM-TGATTCTCAGCCCTTCGC- BHQ1-3′). For TaqMan RT-PCR, the 25 µL reaction contained 1X Reaction mix of SuperScriptIII Platinum One-Step Qrt-PCR System (Invitrogen), 0.5 µL of Taq mix, 1 µM of MgSO_4_, 0.25 µM of each primer, 0.1 µM of probe and 3 µL of RNA. Reverse transcription was carried out at 50 °C for 30 min, followed by denaturation at 95 °C for 15 min was amplified with 45 PCR cycles at 95 °C (10 s), 55 °C (20 s) and 72 °C (15 s).

Primers and probe for Norovirus 1 and 2 were used as described by da Silva et al. [36]. For Norovirus 1 (GI) sequence of primers and probes used were QNIF4 FW (5′-CGCTGGATGCGNTTCCAT-3′), NV1LC RW (5′-CCTTAGACGCCATCATCATTTAC-3′) and NVGG1 probe (5′-FAM-TGGACAGGAGAYCGCRATCT-TAMRA-3′); for Norovirus 2 (GII) were used: QNIF2 FW (5′-ATGTTCAGRTGGATGAGRTTCTCWGA), COG2 RW (5′-TCGACGCCATCTTCATTCACA-3′) and QNIFS probe (5′-FAM- AGCACGTGGGAGGGGATCG-TAMRA-3′). For TaqMan RT-PCR, the 25 µL reaction contained 1X Reaction mix of SuperScriptIII Platinum One-Step Qrt-PCR System (Invitrogen), 0.5 µL of Taq mix, 1 µM of MgSO_4_, 0.5 µM of each forward primer, 0.9 µM of each reverse primer, 0.25 µM of probe and 5 µL of RNA. Cycling condition was as follows: 60 min at 55 °C, 5 min at 95 °C, and 45 cycles of three step cycling consisting of denaturation at 95 °C for 15 s, annealing at 60 °C for 1 min and extension at 65 °C.

Sterile water was used as negative control and no template control (NTC) in all the PCRs. Positive controls for HAV, Norovirus 1 and Norovirus 2 were provided as plasmids by “Istituto Superior di Sanità” (ISS). In particular, they were identified as: plasmid IC HAV 2014, plasmid IC NoVGI 2014 and plasmid IC NoVGII 2014. The positive control for HEV PCR is a field sample identify by genotyping as genotype 3.

The occurrence of bacterial pathogens was investigated through dilution method. Tap water samples (50 mL) were stored for 24 h (4 °C). After mixing, 1 mL of each sample was taken and used for the preparation of the scalar dilutions in sterile physiological solution up to a dilution of 1:10-10. One mL of each dilution was spread on Columbia Agar Blood (AS), Plate Count Agar (PCA) used for the determination of the total microbial load in water matrices and Oxytetracycline Glucose Yeast Extract Agar (OGYE) for the growth of yeasts and molds. The three media (AS, PCA, OGYE) were incubated in a thermostat at both 20 ± 2 °C and 37 ± 2 °C for 72 h and checked daily. Colonies were cloned by type of morphology on AGS and classified by phenotypic and biochemical tests (API 20 E and 20 NE). Analyses were performed in triplicates.

### 2.5. Determination of Cyanotoxins

Microcystin/Nodularin, Anatoxin A, Cilindrospermopsin and β-N-methylamino-L-alanine (BMAA) were determined in tap water from both sites. For this purpose, commercial kits based on the ELISA method (Abraxis-Pennsylvania-USA) were used. The method used is that described by the manufacturer for each substance examined. Briefly, Microcystin/Nodularin test is an indirect competitive ELISA for the congener-independent detection of Microcystins and Nodularins. It is based on the recognition of Microcystins, Nodularins, and their congeners by specific antibodies. Toxin, when present in a sample, and a Microcystins-protein analogue immobilized on the plate compete for the binding sites of the anti-Microcystins/Nodularins antibodies in solution. The plate is then washed and a second antibody-HRP label is added. After a second washing step and addition of the substrate solution, a color signal is generated. The intensity of the blue color is inversely proportional to the concentration of Microcystins present in the sample. The detection limit (LOD) for this assay was 0.10 μg/L.

The Anatoxin-a test is a direct competitive ELISA based on the recognition of Anatoxin-a by a monoclonal antibody. Anatoxin-a, when present in a sample, and an Anatoxin-a-enzyme conjugate compete for the binding sites of mouse anti-Anatoxin-a antibodies in solution. The Anatoxin-a antibodies are then bound by a second antibody (anti-mouse) immobilized on the microtiter plate. After a washing step and addition of the substrate solution, a color signal is generated. The intensity of the blue color is inversely proportional to the concentration of Anatoxin-a present in the sample. The LOD for this assay was 0.10 μg/L.

Cylindrospermopsin test is a direct competitive ELISA for the detection of Cylindrospermopsin. It is based on the recognition of Cylindrospermopsin by specific antibodies. Cylindrospermopsin, when present in a sample, and a Cylindrospermopsin-HRP analogue compete for the binding sites of rabbit anti-Cylindrospermopsin antibodies in solution. The anti-Cylindrospermopsin antibodies are then bound by a second antibody (goat anti-rabbit) immobilized on the wells of the microtiter plate. After a washing step and addition of the substrate solution, a color signal is generated. The intensity of the blue color is inversely proportional to the concentration of Cylindrospermopsin present in the sample. The LOD for this assay was 0.040 μg/L.

BMAA test is a direct competitive ELISA based on the recognition of BMAA by specific antibodies, when present in a sample, and a BMAA-HRP analogue competes for the binding sites of the rabbit anti-BMAA antibodies in solution. The BMAA antibodies are then bound by a second antibody (goat anti-rabbit) immobilized on the wells of the microtiter plate. After a washing step and addition of the substrate solution, a color signal is generated. The intensity of the blue color is inversely proportional to the concentration of BMAA present in the sample. The LOD for this assay was 4 ng/mL.

For all tests, the color reaction is stopped after a specified time and the color is evaluated using an ELISA reader (Erba Lisa Scan II, ERBA Diagnostics Mannheim, Mannheim, Italy). The concentrations were determined by interpolation using the standard curves constructed with each run. Analyses were performed in triplicates.

### 2.6. Determination of Endocrine Interference Activity with CALUX Method

For CALUX (Chemical Activated LUciferase gene eXpression) test, 250 mL of water sample was transferred to a 500 mL bottle and added with 50 mL of ethyl acetate, then sample was placed on shaker for 30 min at 200–240 rpm. The organic phase was transferred to a glass test tube. The extraction cycle was repeated twice. The extract was subjected to slight heating and a low nitrogen flow. The pellet was resuspended in 40 μL of DMSO. CALUX assay was performed as described in Standard Operating Procedures (BioDetection System, Amsterdam, The Netherlands). The Estrogenα Responsive (ERα) CALUX (BioDetection Systems, Amsterdam, The Netherlands) was generated from a human bone cell line (U2-OS) by stable transfection with an EREs (estrogen responsive elements) expression plasmid and a luciferase reporter construct. Modified U2-OS were cultured in DMEM/F-12 (Dulbecco’s Modified Eagle Medium/Nutrient Mixture F-12) medium supplemented with 7.5% Foetal Calf Serum (FCS). U2OS cell line was seeded in 96-well plates with DMEM/F-12 medium phenol red-free added with 5% of DCC-FCS (dextran-coated charcoal-stripped FCS, 200 µL per well). After 24 h, the medium was replaced with exposure medium, then a standard curve consisting of ten points of the reference molecule for Erα- CALUX, (provided by BioDetection System, Amsterdam, The Netherlands) was added. In the remaining wells, the cells were incubated with the extracts dissolved in DMSO. Each point of the reference curve was analyzed in triplicate, each extract was diluted twice (3X and 10X) and loaded in triplicate. After 24 h of exposure at 37 °C and 5% CO_2_, the medium was removed and the cells lysed, then the luciferase activity was measured using a luminometer (Berthold Tristar 2). The standard curve was fitted using the sigmoidal fit in a spreadsheet developed and provided by BioDetection Systems (Amsterdam, the Netherlands). The amount of luciferase produced by the samples was related to known concentrations of reference compound and the results are therefore expressed as reference compound.

### 2.7. Solid-Phase Extraction of Water Samples

Water concentrates were obtained following the solid-phase extraction based on “US Environmental Protection Agency 525.2 Method” [37,38]. A 10 g/60 mL trifunctional C18 silica column (Strata C18-E, 55 µm, 70 A, 10 g/60 mL, Phenomenex) connected to the Visiprep system, which allows the passage of aqueous sample through the solid phase by means of the vacuum applied by a diaphragm pump, was used. Ten milliliters of 95% sulfuric acid (H_2_SO_3_) were used to acidify the water and allow the adsorption of both neutral and acidic compounds. Dichloromethane, ethyl acetate and methanol (40 mL) have been sequentially added to the column and a flux speed was set at 20 mL/min for 28 h. Column was air-dried for 6 h. For the elution, 40 mL of dichloromethane, ethyl acetate, and methanol were used. The eluate was evaporated (45 °C) with a rotavapor. The pellet containing the water concentrates was resuspended in 5 mL of dimethyl sulfoxide (DMSO), resulting in a concentrates dilution of 1:20.

### 2.8. Toxicity Assay on EPC Cell Line Monolayers

Prior sublethal toxicity experiment, the integrity of the EPC cell monolayer exposed to DMSO and water concentrates from sites A and B was evaluated [21]. Scalar 1:2 dilutions were made starting from pure DMSO and concentrate stock solutions. The toxic effect of each dilution was assessed in triplicate. The cell monolayers were checked daily for 10 days to determine the higher dilution in which the cytotoxic effect was observed. These sublethal dilutions (1:80, 1:160, 1:640 for DMSO, site A, and site B, respectively) were then used to assess the oxidative pressure driven by water concentrates, in order to compare the effects of water samples when cytotoxicity is normalized in cell line. Twenty-one flasks 75 cm^2^ (60 mL) were prepared for each water sample, including a negative (PBS) and positive (DSMO) control. The flasks were inoculated with 1.3 mL of sample and placed in an incubator at 15 °C. Each treatment was performed in triplicate and seven endpoints (0, 6, 12, 24, 48, 72 and 96 h from inoculation) were set. Detachment of cells was carried out with the addition of 10 mL of PBS and then centrifuged at 1000 rpm for 15 min at 20 °C. Each cell pellet was used for biochemical analysis.

Biochemical analyses in EPC cell line were conducted according to previous studies on cell lines [21,39,40]. In order to extract the cytosolic fraction, EPC cell pellet was washed with phosphate-buffered saline (PBS) 1X and centrifuged at 130× *g* (5 min, 4 °C) for three-fold. The resulting pellet was resuspended in a sodium phosphate buffer (NaH_2_PO_4_ + Na_2_HPO4) 50 mM pH 7.4, NaCl 200 mM, bacitracin 0.1 mg/mL and aprotinin 0.008 tiu/mL. EPC was sonicated three-fold for 15 s and then centrifuged at 20,000× *g* (10 min, 4 °C). The activity of antioxidant enzymes was measured in triplicate by spectrophotometry (Varian Cary 100) at 25 °C. The SOD levels were assessed at 550 nm in 50 mM Na_2_CO_3_ buffer pH 10 with 0.1 mM EDTA, 500 mM cytochrome C, 1 mM hypoxanthine, and xanthine oxidase. The CAT activity was performed at 240 nm following the consumption of H_2_O_2_ using sodium phosphate buffer 100 mM pH 7 and 24 mM H_2_O_2_. The GPx activity was measured at 340 nm in sodium phosphate buffer 100 mM pH 7.5 added with EDTA 1 mM, NADPH 0.12 mM, GSH 2 mM, GR 1U and 0.6 mM H_2_O_2_. The GST activity was determined at 340 nm in sodium phosphate buffer 100 mM pH 6.5 using GSH and 1-chloro-2,4-dinitrobenzene (CDNB) 2 mM. The GR assay was performed at 340 nm in sodium phosphate buffer 100 mM pH 7 using GSSG 1 mM and NADPH 0.06 mM. The cytosolic total protein concentration was determined according to Lowry et al. [41]. IBRv2 method was used to integrate the biomarkers’ responses, allowing the comparison between the two survey sites. Data of Site A and B were normalized with that of the DMSO control group.

### 2.9. Statistical Analyses

Normality and homogeneity of variance were tested using the Shapiro–Wilk test. Differences in concentration of the physicochemical features between the two sites were checked using the Wilcoxon test since the null hypothesis for the homogeneity of variance and/or for normal distribution could not be rejected. For oxidative stress biomarkers, one-way ANOVA following Tukey’s post–hoc test was used to check statistically significant differences (*p* < 0.05) between the experimental groups at the same time point. Statistical analyses were performed using RStudio^®^ version 1.1.463 (RStudio, Boston, MA, USA).

## 3. Results

### 3.1. Water Physicochemical Parameters

Table 1 presents the mean values and standard deviations of the physicochemical features measured at the two sites (three replicates for each parameter). Significant differences between the sites were found only for conductivity (*p* < 0.01).

### 3.2. Metals in Water

All the analyzed water samples were found under the LOQ and compliant with the maximum limits (LMs) for the indicator parameters in water for human consumption set by the Legislative Decree No. 31 of the Italian Government of 02/02/2001 (Legislative Decree No. 31 implementing Directive 98/83/EC on the quality of water intended for human consumption).

### 3.3. Viral and Bacterial Pathogens in Water Samples

All the samples analyzed were negative for the detection of the three viral pathogens Hepatitis A virus, Hepatitis E virus and Norovirus 1 and 2. After 72 h of incubation at both temperatures, no samples showed bacterial, fungi, or yeast growth.

### 3.4. Determination of Cyanotoxins in Water Samples

The results obtained for all the determinations fall within the LOD/LOQ of the standards used for the calibration straight line of the kits; therefore, all samples are considered negative.

### 3.5. Determination of Endocrine Interference Activity

**Figure 1:**
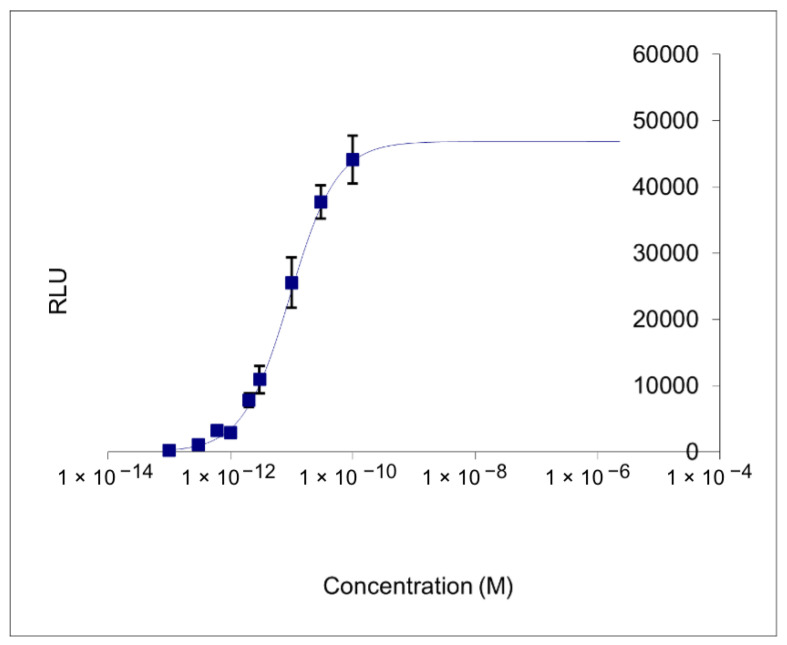
Calibration curve of ER-Calux^®^ bioassay. Graphical representation of the results from the nine calibrators. RLU (relative light unit) are units used for the luminescent measurement.

All the water samples analyzed were negative for estrogenic activity (Figure 1, Table 2).

### 3.6. Oxidative Stress Responses in EPC Cell Line

**Figure 2:**
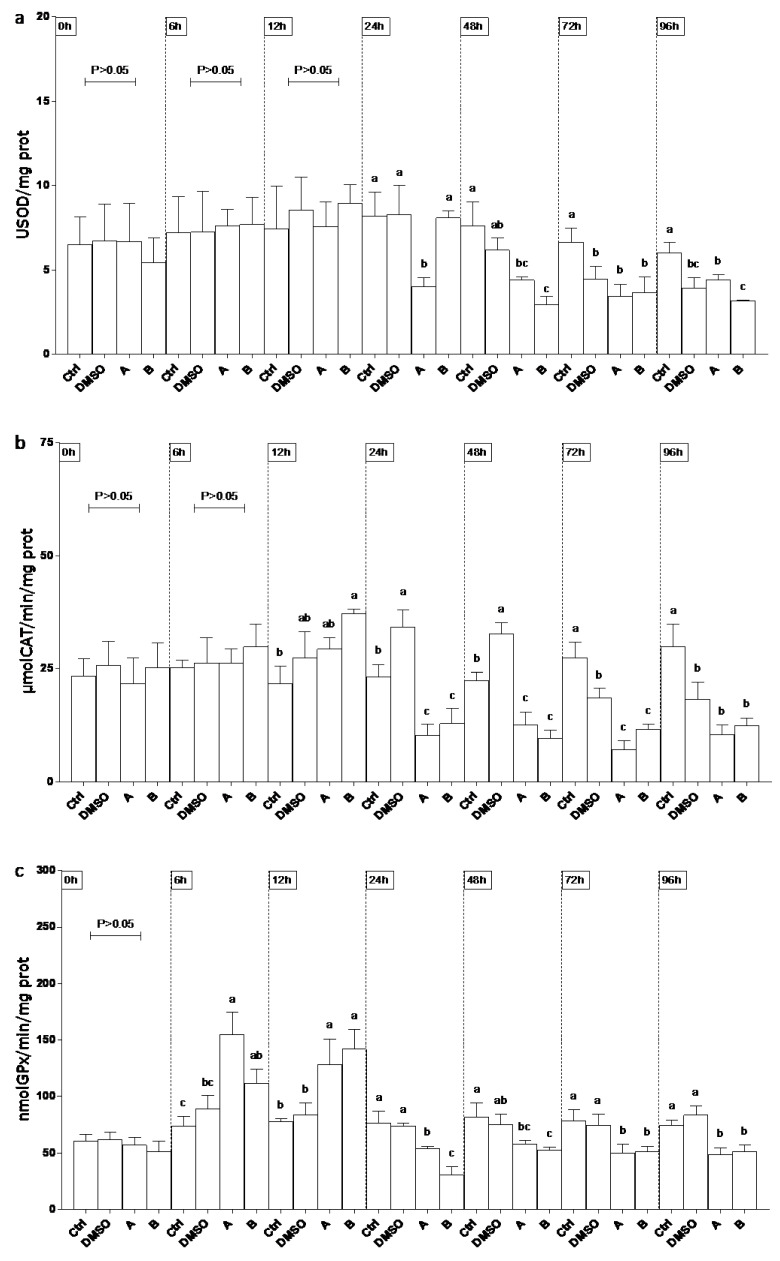
Superoxide dismutase (SOD; (**a**)), catalase (CAT; (**b**)) and glutathione peroxidase (GPx; (**c**)) activity in epithelioma papulosum cyprini (EPC) cell line exposed to concentrated drinking water from sites A and B. Ctrl denotes control. Different lowercase letters indicate statistically significant differences (*p* < 0.05) between the two groups at the same time point.

**Figure 3:**
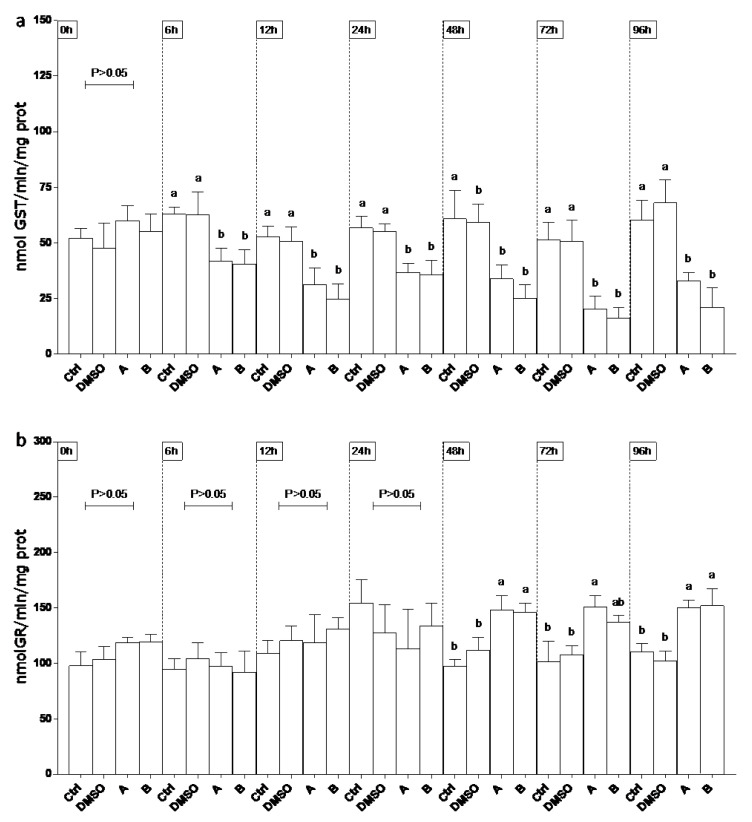
Glutathione S-transferase (GST; (**a**)) and glutathione reductase (GR; (**b**)) activity in epithelioma papulosum cyprini (EPC) cell line exposed to concentrated drinking water from sites A and B. Ctrl denotes control. Different lowercase letters indicate statistically significant differences (*p* < 0.05) between the two groups at the same time point.

SOD activity (Figure 2a) was lower (60%) than controls (Ctrl and DMSO) in site A on 24 and 48 h, whereas on 72 and 96 h significant decrease was observed only compared to Ctrl. At 48 h SOD levels were found lower than Ctrl (70%) and DMSO (50%) in site B. At the last experimental endpoints (72 and 96 h) enzyme lowered in DMSO to the levels of group A and B. CAT levels (Figure 2b) showed a biphasic trend in the DMSO group during the experimental period. At 24, 48, and 72 h enzyme activity on sites A and B significantly decreased (70%) compared to both controls. On 96 h, CAT activity at sites A and B remained lower than Ctrl. An early increase of GPx level (onefold, 6–12 h) was observed mainly in both sites, following a decrease (50%) compared to DMSO (Figure 2c). A consistent lower activity of GST (50%) was measured through the experiment in all sites compared to Ctrl and DMSO (Figure 3a). GR activity significantly increased (80%) compared to both control groups in sites A and B (Figure 3b).

### 3.7. Integrated Biomarker Responses Version 2 (IBRv2)

**Figure 4:**
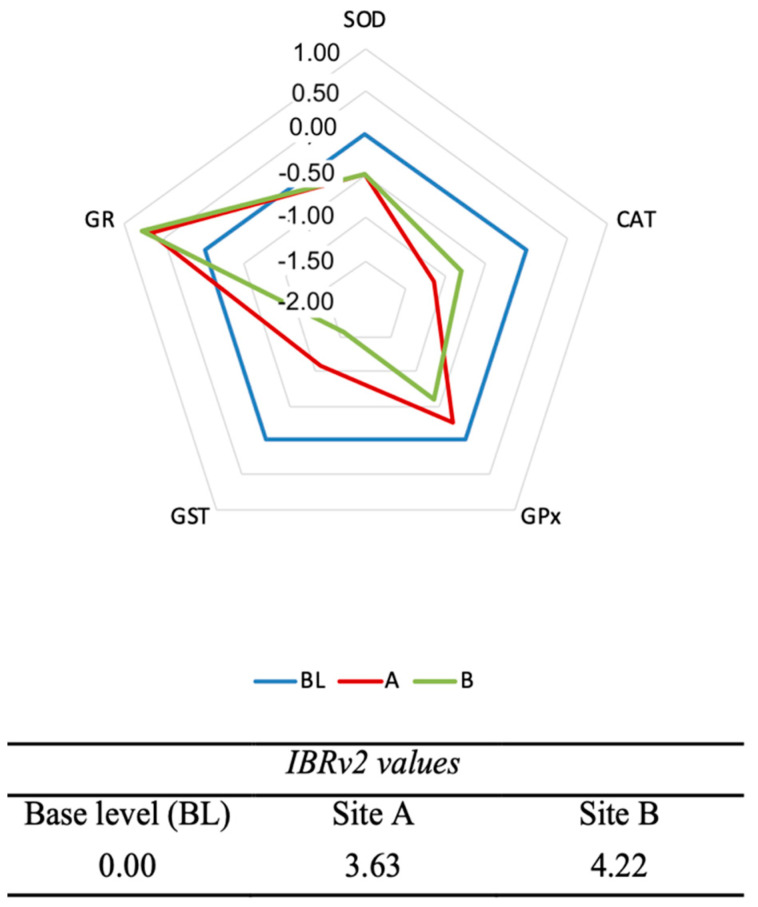
Star plot and IBRv2 values in epithelioma papulosum cyprini (EPC) cell line exposed to concentrated drinking water from sites A and B. CAT denotes catalase, GPx glutathione peroxidase, GR glutathione reductase, GST glutathione S-transferase, SOD superoxide dismutase.

The results of IBRv2 on oxidative stress responses in EPC cell line are reported in Figure 4. The star plots show the overall contribution of the activity of each enzyme during the whole experimental period in both in vitro and in vivo systems exposed to water concentrates from the two sites. The data of each municipality were compared and normalized with that of the DMSO control, which in the star plot represents level 0. The star plots indicate a general failure of the enzymatic activity of SOD, CAT, GPx, and GST in the EPC cell line. IBRv2 values for sites A and B were 3.63 and 4.22, respectively.

## 4. Discussion

Changes in physical-chemical parameters, such as temperature, pH, salinity, and chemical elements such as dissolved oxygen, chemical oxygen demand, nitrate, and phosphate, are recognized as valuable indicators of water quality, providing a reliable scenario of the health status of water body [42]. Another indicator of good water quality is the presence of metals. In aquatic ecosystems, trace elements can deteriorate the environment, causing severe ecological threats following toxicity for the exposed organisms. Essential metals in trace quantities are necessary to exploit physiological and biochemical functions in organisms, whereas non-essential heavy metals (Cd, Pb, and Hg) may exert toxicity at low concentrations. Heavy metals are reported as carcinogenic, mutagenic, and teratogenic, leading to the generation of reactive oxygenic species (ROS) and oxidative stress. This harmful condition can also be spread through the food chain when metals cannot be metabolized by organisms, following biomagnification [43]. Moreover, high levels of toxic metals can pose a risk for drinking, aquaculture, and livestock production [44,45,46]. Water can also host a large variety of pathogens depending on the environmental source. In groundwater, a wide range of pathogens are known, such as Hepatitis A and E virus, *Escherichia coli*, *Salmonella* spp., *Shigella* spp. and *Vibrio cholerae* [47]. In the present study, physical-chemical parameters and trace elements were lower than the limit threshold imposed by law in both sampling sites (Council Directive 98/83/EC of 3 November 1998 on the quality of water intended for human consumption; Official Journal L 330, 05/12/1998 P. 0032-0054). These findings suggest good quality of tap water in sites A and B. The absence of pathogens positivity found either for bacteria, fungi, and yeasts nor for viruses in chlorine-treated groundwater from both sites corroborates this hypothesis.

Cyanotoxins are naturally present in surface waters, but may be transferred to groundwater through hydrologic processes, resulting in water contamination [48,49]. They have been associated with harmful effects, providing toxicity to plants, animals, and humans [50]. The absence of cyanotoxins in chlorine-treated groundwater from sites A and B confirms water quality and safety for humans, animals, and aquatic organisms, and ecosystems.

Ecotoxicologically relevant concentrations in waters of organic micropollutants such as pharmaceutical products can reach the environment leading to a threat to drinking water quality and the aquatic ecosystem. Endocrine active substances (EAS) are synthetic hormones with a high bioactive potential affecting the endocrine system of organisms even at very low concentrations [51]. Previous study showed a concentration of EAS up to 100 ng/L in surface water, causing endocrine-related adverse health effects in wildlife and human [52,53,54]. These findings are in contrast with our results in groundwater, again suggesting a good quality of water from sites A and B.

In this study, the EPC cell line was a sensitive experimental model assessing the aquatic toxicity of water concentrates, providing valuable information on the potential long-term animal and environmental risks. Previous studies have shown that the results on cell lines of aquatic organisms treated with emerging pollutants were comparable with those obtained in in vivo systems, indicating that cell lines are a rapid, reproducible, and sensitive tool for investigating the toxicological effects of environmental contaminants [55]. Moreover, the treatment of freshwater organisms with high but not lethal concentrations of xenobiotics is a suitable experimental approach to reproduce the biochemical-molecular stress responses of long-term exposure in a shorter experimental period [24]. Since water from sites A and B showed similar chemical-physical parameters and the absence of contamination by metals, pathogens, cyanotoxins, and endocrine active substances, we can assume that the modulation of antioxidant responses in EPC cell line may be attributed to chlorine-based biocides and related DBPs. IBRv2 showed a similar muted risk for aquatic organisms exposed to water concentrates from A and B. A late decrease in SOD activity was observed in both investigated sites. This outcome is in agreement with the previous study of Wang et al. [56] in embryos of zebrafish *D. rerio* exposed to halobenzoquinone (HBQ), an emerging DBPs of the chlorination process. The failure of SOD activity was associated with a significant increase in ROS, suggesting a severe oxidative pressure [56]. IBRv2 also indicated that the contribution of CAT and GPx against the oxidative stress induced by water concentrates was similar for in vitro system, with a more marked decrease in CAT levels in site A and on GPx in site B. Our results are in line with a previous study on *Cyprinus carpio* exposed to sodium hypochlorite (NaClO) and chlorine dioxide (ClO_2_), two of the most used biocides in the chlorination process [16]. The authors supposed that the biocides or related DBPs generated during the experimental exposure may have enhanced the prooxidant mechanisms in treated fish [16]. Data obtained in the present study can support this hypothesis for fish. The braking of the GPx activity, which is a fundamental defense line against oxidative damage, may be related to an intracellular variation in the levels of reduced glutathione (GSH). GSH is one of the most powerful antioxidant molecules and is ubiquitously distributed through the organisms. It can promote oxidative defense directly or as a cofactor of antioxidant and detoxifying enzymes, such as GPx and GST [57]. In the present study, we did not measure the GSH levels, but a previous mechanistic study showed that the DBP 3-Chloro-4-(dichloromethyl)-5-hydroxy-2(5H)-furanone can drop the levels of GSH, following oxidative damages as proved by the increased levels of malondialdehyde and DNA damage [58]. This evidence was also confirmed by Wang et al. [56] in *D. rerio* embryos exposed to HBQ. Indeed, the GR activity measured in the present study can be considered a good indicator of GSH regeneration. The increase of GR levels, along with the time-course GPx and GST levels, may suggest a slightly efficient regeneration of GSH, which was sufficient to support only the early activity of GPx. It is well known that exposure to DBPs, such as dichloroacetic acid (DCA), can result in a boost in the activity of GST in order to metabolize them [2,16,59]. In the present study, we observed contrasting results compared to previous studies on *C. carpio*, in which an enhancement of enzyme activity was measured following chlorine biocides treatment [2,16].

## 5. Conclusions

In conclusion, chemical-physical parameters, metals levels, the absence of pathogens, cyanotoxins and endocrine active substances, and oxidative stress biomarkers response highlighted a low risk in sites A e B of North Italy. The experimental approach provided useful information about the water quality and animal and human health risks elicited by groundwater.

## Figures and Tables

**Table 1:** Mean and standard deviation (three replicates) of physicochemical parameters and nutrients measured at site A and site B. NO_3_^–^ denotes nitrate, NH_4_^+^ ammonia, PO_4_^3–^ phosphate concentrations (mg L^−1^).

Parameter	Site A	Site B
pH (unit)	8.32 ± 0.12	8.02 ± 0.09
Conductivity (μS cm^−1^)	132 ± 3.14	226 ± 2.34
NH_4_^+^ (mg L^−1^)	0.04 ± 0.001	0.03 ± 0.002
NO_3_^−^ (mg L^−1^)	8.45 ± 1.24	9.23 ± 2.15
PO_4_^3−^ (mg L^−1^)	0.01 ± 0.01	0.02 ± 0.01

**Table 2:** Results of the two water samples with ER-Calux^®^ bioassay. DMSO denotes dimethyl sulfoxide, M concentration SD standard deviation.

	Extraction Information			Bioassay Calculation			Summary Results
Sample	Extracted	Unit	DMSO (uL)	Dilution	M	SD (%)	17ß-Estradiol Eq.
**A**	250	mL	40.00	1	<Min	1.5	<Min
**A**	250	mL	40.00	3	<Min	1.2	<Min
**A**	250	mL	40.00	10	<Min	1.5	<Min
**B**	250	mL	40.00	1	<Min	2.7	<Min
**B**	250	mL	40.00	3	<Min	2.3	<Min
**B**	250	mL	40.00	10	<Min	0.0	<Min
**DMSO**	2.00	mL	40.00	1	<Min	0.5	<Min

## Data Availability

Not applicable.
